# Assessment of optometrists' referral accuracy and contributing factors: A review

**DOI:** 10.1111/opo.13183

**Published:** 2023-07-03

**Authors:** Josie Carmichael, Sarah Abdi, Konstantinos Balaskas, Enrico Costanza, Ann Blandford

**Affiliations:** ^1^ University College London Interaction Centre (UCLIC), UCL London UK; ^2^ NIHR Biomedical Research Centre at Moorfields Eye Hospital NHS Foundation Trust and UCL Institute of Ophthalmology London UK

**Keywords:** false‐positive, optometrists, optometry, referral accuracy, referrals

## Abstract

**Purpose:**

In the UK, ophthalmology has the highest number of outpatient appointments within the National Health Service. False‐positive referrals from primary care are one of the main factors contributing to the oversubscription of hospital eye services (HESs). We reviewed the accuracy of referrals originating from primary care optometrists and contributing factors, such as condition type and years since registration.

**Recent findings:**

Of the 31 studies included in the review, 22 were retrospective analyses of referrals and appointments at the HES. Eight were prospective studies, and one used online clinical vignettes. Seven assessed the accuracy of referrals for all ocular conditions. The remaining studies focused on glaucoma (*n* = 11), cataracts (*n* = 7), emergency conditions (*n* = 4), neovascular age‐related macular degeneration (*n* = 1) and paediatric binocular vision (*n* = 1). The diagnostic agreement for suspected emergency ocular conditions was the lowest, with only 21.1% of referrals considered to require urgent attention in one study. For glaucoma, the first‐visit discharge rate was high (16.7%–48%). Optometrist referral accuracy was overall 18.6% higher than General Medical Practitioners'; however, the two mainly referred different ocular conditions. Female optometrists made more false‐positive referrals than males (*p* = 0.008). The proportion of false positives decreased by 6.2% per year since registration (*p* < 0.001).

**Summary:**

There was significant variation in referral accuracy across different ocular conditions, partly due to differences when defining accurate referrals. Optometrists working in primary care are generally more limited in their resources than the HES. Thus, choosing the cautious option of referral when they are unsure could be in the patients' best interests. The possible effect of increased use of advanced imaging on referrals requires evaluation. Although interventions such as refinement schemes have been put in place, these vary across regions, and their approaches such as virtual referral triaging may reduce unnecessary HES face‐to‐face appointments and promote communication between primary and secondary care.

**Key points**

- Studies have reported a large variation in referral accuracy across ocular conditions, partly due to differences in criteria used to define an accurate referral.
- A shorter time since qualification was associated with a higher proportion of false‐positive referrals made by optometrists, likely due to less experienced clinicians making more cautious clinical management decisions.
- Efforts have been made to reduce the false‐positive referrals entering the hospital eye service, but with eyecare pathways varying between regions, it is difficult to determine the best approach.

## INTRODUCTION

Ophthalmic services in the UK are currently under a huge amount of stress. In 2019/2020, ophthalmic departments in the UK had a staggering 7.9 million outpatient attendances,^1^ the highest number of any specialty within the National Health Service (NHS).^1^ Furthermore, ophthalmic service provision capacity is limited by a low number of ophthalmologists per capita,^2^ and the backlog of appointments resulting from the COVID‐19 pandemic has increased the workload for NHS services. This is not just putting a strain on ophthalmic services but can cause delayed appointments for patients needing close monitoring and treatment, at a detriment to their prognosis. A study carried out in the UK reported that delayed follow‐up appointments beyond the clinically recommended interval was the cause of vision loss in 80% of affected patients.^3^

In the UK, the majority of referrals into the hospital eye service (HES) originate from optometric examinations in primary care, with one study carried out in Bradford, UK, finding this proportion to be 72%.^4^ The General Optical Council (GOC) standards of practice guidelines state that optometrists should ‘recognise and work within the limits of their scope of practice’ and ‘be able to identify when they need to refer a patient in the interests of the patient's health and safety, and make appropriate referrals’;^5^ thus, optometrists should refer any condition that they feel unable to manage in practice. However, it is thought that a large number of optometrists' referrals can be considered ‘false‐positives’, meaning that these patients could safely be managed in primary care.^6^, ^7^ This is often reported as a contributing factor to the oversubscription of hospital eye clinics and several studies have assessed the accuracy of referrals for various eye conditions. However, until now, no in‐depth review of referral accuracy from optometrists or the factors that may affect this has been conducted.

In this review, we aim to evaluate the accuracy of referrals originating from primary care optometric practices as well as the factors that may contribute to optometrists' level of accuracy.

### Objectives

This review has the following specific objectives:

1. To synthesise studies assessing the accuracy of referrals from primary care optometric practices to the HES across different countries.
2. To assess for which ocular condition(s) referrals are the most and least accurate.
3. To identify the factors which may affect the accuracy of referrals from optometrists into the HES.

## METHODS

### Registration

The international prospective register of systematic reviews (PROSPERO) was used to register our review protocol (registration number: CRD42022328721) to prevent duplication and to increase the transparency of our review process.

### Eligibility criteria

To complete a robust systematic search and selection of studies, a checklist of inclusion and exclusion criteria was created. This was to ensure consistency when screening articles and to act as a reference point when making decisions about whether to include/exclude articles. The decision was made to exclude studies that assessed referrals from diabetic retinopathy screening programmes. This decision was made as although many optometrists work as diabetic screening graders and make referral decisions, this pathway does not represent the typical referral pathway from primary care optometry practices. Table 1 summarises the inclusion and exclusion criteria checklist, respectively. Articles were screened for their suitability against these criteria.

**TABLE 1:** Summary of the inclusion/exclusion criteria.

Criteria	Inclusion	Exclusion
Time period	December 2001–December 2022	Prior to Dec 2001
Language of study	English	Any other language
Study design	Quantitative studies of current practice including (but not limited to): controlled, uncontrolled studies, surveys, retrospective analysis, clinical vignettes	Qualitative or mixed methods studies. Interventions in pilot studies, viewpoints, editorials, conference/meeting abstracts, expert opinions and grey literature. Systematic or similar reviews (e.g., narrative, scoping and realist reviews)
Setting	Any setting involving primary eye care	Internal referrals within secondary care, General Medical Practitioner (GP) referrals
Participants	Studies focussing on primary care optometrists making referrals to secondary care	Studies focussing on referrals from GPs, other allied health professionals or patients who self‐refer (e.g., patients attending Accident and Emergency without a recommendation from an optometrist)
Condition focus	Any eye condition or conditions, which have been referred to the hospital (can include anterior and posterior eye conditions)	Referrals by optometrists to non‐hospital eye service (HES) departments due to systemic conditions showing signs in the eye (e.g., referral to GP for blood pressure check). Referrals from diabetic retinopathy screening programmes
Topic focus	Quantitative assessment of: The % or number of referrals that are correct/incorrect from optometrists The individual factors affecting the accuracy of referrals from optometrists	Assessment of referral letter quality Assessment of the source of referral, for example, ‘of all glaucoma referrals, 80% come from optometrists’ but no assessment of whether these are correct/incorrect Studies that have not assessed referrals from optometrists separate from other sources, that is, all referrals from primary care are assessed

We included primary studies that used a quantitative design and were written in English. We did not exclude studies based on our assessment of methodological limitations but used the information about methodological limitations to assess our confidence in the findings. We excluded abstracts without a corresponding full paper, as they were unlikely to provide sufficiently rich data.

### Search strategy

PRISMA was used to help guide our protocol development.^8^ PUBMED, MEDLINE and CINAHL were searched for potential studies for inclusion. Initially, a search was also performed using Google Scholar; however, this returned a large number of irrelevant results, with relevant papers being duplicated from the other databases. We developed search strategies for the databases. Studies published during or after December 2001 were included to ensure an assessment that is representative of recent practice. Table 2 presents the final facets and keywords used when searching databases. In addition to database searching, we reviewed the reference lists of all included studies and other key references, which allows a method of ‘reference chaining’.

**TABLE 2:** Facet terms and their keywords used for database searching.

Number assigned to facet	Facet	Keywords	Boolean
1	Optometrist	1. Optometrist(s) OR 2. Optometry OR 3. Primary eye care OR 4. Primary eye clinic(s) OR 5. Optician(s)	1 AND 2
2	Referral practice	1. Referral(s)	

### Selection process

All articles identified from database searches were organised in EndNote and duplicates were removed. The primary researcher (JC) conducted screening of the title and abstracts of all search results. A second researcher (SA) also screened all titles and abstracts. An initial sample of 20% was first screened by both researchers to assess agreement. All articles where the researchers disagreed were reviewed together and differences in interpretation of the inclusion/exclusion criteria were discussed. The remaining studies (80%) were screened by both researchers independently with a good level of agreement (κ = 0.82). Studies where the two reviewers disagreed were discussed and a decision was reached to include/exclude each one. After the screening phase, 76 studies met the criteria for full‐text assessment.

The full texts of all 76 studies were assessed by the primary researcher. The secondary researcher screened the full text for a sample of 20%, and agreement was checked. At this stage, there was a 93.3% agreement rate between the two reviewers. For one study, the reviewers initially disagreed, but after a discussion based on the inclusion/exclusion criteria they agreed that the study should be excluded.

### Data collection and items

Data collection was carried out by one reviewer (JC) who worked independently. Prior to collection, a form was designed to extract all relevant data from each included study. This form was part of a study protocol that was written by JC and reviewed by SA and AB prior to data extraction. The form included information regarding sample characteristics, objectives, study design, data collection and analysis methods, quantitative findings, conclusions, limitations and any relevant tables, figures or images. Table 3 summarises the information extracted from each article.

**TABLE 3:** Information extracted from all studies included in the review.

Information extracted	
1	Author(s)
2	Year
3	Title
4	Country
5	Study aim(s)
6	Study design
7	Sample period
8	Sample size
9	Eye condition(s)
10	Method used to determine referral accuracy
11	Main results
12	Limitations
13	Other important findings

### Quality assessment

In this review, we focus on papers that are the most relevant, rather than papers which meet a specific standard of methodological quality. We aimed to exclude studies only if they were considered ‘fatally flawed’, for example, the research design was not clearly specified; however, no relevant studies were deemed as such. This has previously been described as prioritising ‘signal’ over ‘noise’^9^ and aims to maximise the inclusion of relevant papers which can add valuable insights. Rather than excluding studies based on quality, they were included but critiqued during review to ensure transparency.^10^ When critiquing study quality, we mainly focussed on sample size for referrals, number of optometrists from which the referrals originated, number of practices from which the referrals originated, study design with respect to prospective or retrospective analysis and the appropriateness of any statistical methods that were used.

### Synthesis of results

A narrative review approach was taken when synthesising the results. This was chosen as we wanted to provide a detailed assessment of studies reporting quantitative accuracy of optometric referrals, while keeping an exploratory approach. We aimed to keep our research question broad with respect to study focus variation and definitions used across the studies. We summarised the accuracy of referrals with an emphasis on referral necessity and divided the analysis into ocular conditions to identify any areas in which improvement in patient management is most evidently needed.

The Economic and Social Research Council (ESRC) developed guidance on the conduct of narrative syntheses.^11^ This was referred to when carrying out this review to increase transparency and trustworthiness. The framework consists of four elements:

1. Developing a theory of how the intervention works, why and for whom.
2. Developing a preliminary synthesis of findings of included studies.
3. Exploring relationships within and between studies.
4. Assessing the robustness of the synthesis.

## RESULTS

### Study selection

**FIGURE 1:**
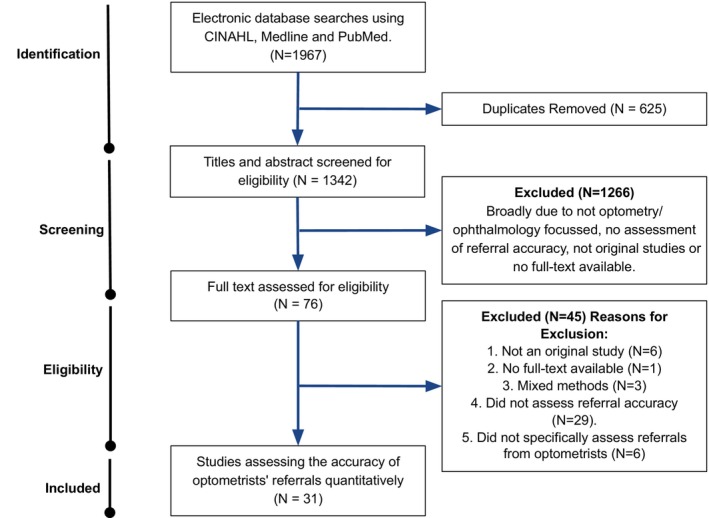
The Preferred Reporting Items for Systematic Reviews and Meta‐Analyses (PRISMA) flow chart detailing the selection process for the studies reviewed.

Thirty‐one studies were selected for analysis. The results from the search and selection process are shown in Figure 1.

### Study characteristics

Of the 31 studies included in the review, 22 were retrospective analyses of referrals and clinical visits to the HES, eight were prospective studies of referrals^12^, ^13^, ^14^, ^15^, ^16^, ^17^, ^18^, ^19^ and one study used online clinical vignettes.^20^ Seven studies reported results from statistical testing, with six using *p*‐value testing for significance^4^, ^20^, ^21^, ^22^, ^23^, ^24^ and one study using kappa agreement.^25^ Studies varied in terms of length, number of referrals, country, definition of accurate referral/true positive referral and the ocular condition(s) assessed. Details of the studies can be found in Tables 4, 5, 6, 7, 8, 9, 10, 11, 12.

**TABLE 4:** Studies assessing referrals for all ocular conditions.

Study	Year	Country	Study design	Study period	Number of referrals	Definition for correct/incorrect	Results
Evans et al.^29^	2021	UK	Retrospective review of referrals. Three dyads of optometry practice and HES in England	May 2015–January 2018 (2 years, 7 months)	459	Researcher opinion: Whether the referral was to an appropriate professional Whether the referral was necessary Whether the referral was accurate	Referrals to an appropriate professional 95.6%–100% Referral necessary 92.9–96.7 Referral accurate 81.1%–97.5%
Shah et al.^30^	2021	UK	Retrospective review of referrals. Six dyads of optometry practice and HES in England and Scotland	May 2015–January 2018 (2 years, 7 months)	905	Researcher opinion: Whether the referral was to an appropriate professional Whether the referral was necessary Whether the referral was accurate	Referrals to an appropriate professional 90.0%–100% Referral necessary 90.8%–97.5% Referral accurate 81.1%–97.5%
Lundmark and Luraas^15^	2017	Norway	Prospective electronic survey	November 2014–December 2017 (3 years, 1 month)	791	Subjective assessment of the concordance of diagnostic codes and texts in referrals and medical reports, made by the two authors together	Primary referral diagnosis matching primary medical report diagnosis 73.8% Mismatched diagnoses 21.1% Incomplete data 5.1%$ Primary referral diagnosis matching primary or secondary medical report diagnosis 79.8% Mismatched diagnoses 15.7% Incomplete data 4.6%
Davey et al.^26^	2016	UK	Retrospective review of referrals (sample of first 30% of new outpatient appointments each month)	December 2007–December 2008 (1 year)	366	True positive: Ophthalmologist confirmed condition/pathology that referrer had stated, where the ophthalmologist's decision to discharge must not have been solely influenced by clinical techniques that were not commonly available to the referring practitioner Diagnostic agreement: Referring Diagnosis agrees with hospital	True positive: 361 (71%) Diagnostic agreement: 244 (67%)
Fung et al.^27^	2016	UK	Retrospective review of referrals	Backdated from 2014 (first quarter) until 1000 were reached (1991–2014)	569	True positive: patients not being discharged from HES with a ‘normal vision’ diagnosis Diagnostic agreement: Concordance in referred condition and diagnosed condition at HES between optometrists and ophthalmologists	True positive: 93.8% Diagnostic agreement: 76.1%
Pierscionek et al.^28^	2009	UK	Retrospective review of referrals	January–March 2007 (3 months)	323	True positive: Patient diagnosed as anything other than ‘no abnormality detected by ophthalmologist’ Diagnostic agreement: Referral diagnosis compared to final diagnosis made by ophthalmologist	True positive: 302 (93.5%) Diagnostic agreement: 225 (69.7%)
Cameron et al.^7^	2009	UK	Retrospective review of referrals	January–June 2005 (6 months)	112	Vetted by six ophthalmologist consultants to classify which referrals required a HES appointment	Required a HES appointment 95 (85%) Did not require HES appointment 11 (10%) GP did not refer onward 6 (5%)

Abbreviation: HES, hospital eye service.

**TABLE 5:** Studies assessing referrals for emergency eye conditions.

Study	Year	Country	Study design	Study period	Number of referrals	Definition for correct/incorrect	Results
Mas‐Tur et al.^25^	2021	UK	Retrospective review of referrals	April 2016–September 2016	1059	Agreement with the assessment by an ophthalmologist but not reliant on equipment available to them	Diagnostic agreement (kappa): Anterior segment 0.87 Vitreo‐retinal 0.68 Medical retina 0.66 Neuro‐ophthalmology 0.59 Glaucoma 0.64 Lids 0.66 Discharged at first visit (54%)
McLaughlin et al.^16^	2018	Canada	Prospective case review	1 April 2016–1 September 2016 (6 months)	57	Alangh's criteria for agreement of diagnosis through categorisation of the provisional diagnosis based on location of pathology Ophthalmologist also determined the urgency of review required	Diagnostic agreement: 30/50 (60%) 7 not yet diagnosed Urgency of review required: Urgent 12 (21.1%) Semi‐urgent 27 (47.4%) Non‐urgent 18 (31.6%)
Nari et al.^39^	2017	Canada	Retrospective review of referrals	17 January 2011–17 July 2011 (6 months)	309	Agreement with the final diagnosis	Diagnostic agreement: Correct 166 (54%) Incorrect 111 (36%) Non‐specific 18 (6%) Not yet diagnosed 12 (4%)
Jackson^34^	2009	Australia	Retrospective review of referrals from two hospitals	Alexandra Hospital 18 April–25 October 2006 (6 months 7 days) Royal Brisbane and Women's Hospital 1 July–30 September 2006 (3 months)	114	Agreement with the diagnosis made in the ophthalmology department	Diagnostic agreement: 55 (48.2%)

**TABLE 6:** Studies assessing referrals for glaucoma.

Study	Year	Country	Study design	Study period	Type of glaucoma	Number of referrals	Definition for correct/incorrect	Correct
Huang et al.^17^	2020	Australia	Prospective review of referrals (control arm)	March 2015–June 2018	All glaucoma referrals	74	Number of referrals resulting in treatment initiation or monitoring on first assessment at the HES	Treatment initiated 25 (33.8%) Monitoring 30 (40.5%) Discharged at first visit 19 (25.7%)
Sii et al.^24^	2019	UK	Retrospective review of referrals pre and post SIGN guidelines	October–November 2014 and September–October 2016	All glaucoma referrals	Pre‐SIGN 312 Post SIGN 325	First visit discharge rate (FVDR)	First visit discharge pre‐SIGN 91 (29.2%) First visit discharge post‐SIGN 63 (19.4%)
Kamel et al.^38^	2019	Republic of Ireland	Retrospective review of referrals and first clinic appointment	January 2007–June 2009 (2 years and 6 months)	All glaucoma referrals	98	Compared to the diagnosis given to each patient during their first assessment at a private eye hospital	Confirmed glaucoma 7 (7%) Glaucoma suspect 14 (14%) Ocular hypertension 11 (11%) Normal 66 (67%) Discharged at first visit 35 (35%)
Annoh et al.^21^	2019	UK	Retrospective review of referrals and first clinic appointment	June–November 2016 (6 months)	Open‐angle and asymptomatic closed angle	715 (95 indicated to have suspect narrow angles)	Clinical diagnosis of PACS, PAC or PACG according to the International Society of Geographical and Epidemiological Ophthalmology classification	False‐positive 36/95 (37.9%) False‐negative 19/715 (3.1%) Discharged at first visit (suspect narrow angles referrals) = 11/95 (12%) Discharged at first visit (overall) 156/715 (25%)
Founti et al.^12^	2018	UK	Multicentre, prospective, observational, cross‐sectional study (however, only in the UK site were there any patients referred by optometrists)	May 2013–March 2014 (10 months)	All glaucoma referrals	28	An outcome was defined as positive when the management plan was an intervention or active monitoring and as negative when the management plan was same‐day discharge	Positive 16 (57.1%, CI 24.6–63%) Negative with same‐day discharge 12 (42.9%, CI 38.8–75.4%)
Khan et al.^40^	2012	UK	Retrospective review of referrals and first clinic appointment	January–March 2011	All glaucoma referrals	102	Compared to the diagnosis given to each patient on their first assessment at the HES	Confirmed glaucoma 17 (17.6%) Glaucoma suspect 18 (17.6%) Ocular hypertension 24 (23.5%) Narrow angles requiring PI 12 (11.8%) No glaucoma or OHT 30 (29%) Discharged at first visit 31 (30%)
Lockwood et al.^14^	2010	UK	Prospective assessment of referrals and clinic appointments	6 months	All glaucoma referrals	441	Compared to the diagnosis given to each patient on their first assessment at the HES	Chronic open angle glaucoma 33 (7.5%) Glaucoma suspect 92 (20.9%) OHT 49 (11.1%) Angle closure glaucoma 8 (1.8%) Pigment dispersion syndrome 1 (0.2%) Trauma 1 (0.2%) Normal 257 (58.3%) Discharged after at least two visits 276 (62.6%)
Ang et al.^23^	2009	UK	Retrospective review of referrals pre and post new GOS contracts	June–November 2005 and June–November 2006	All glaucoma referrals	Old GOS 18: 183 New GOS 18: 120	A true positive was defined as a referral that was found to have definite glaucomatous damage	True positives old GOS 18: 33 (18.3%) True positives new GOS 18: 38 (31.7%) Discharged at first visit old GOS 18: 79 (43.2%) Discharged at first visit new GOS 18: 20 (16.7%)
Salmon et al.^37^	2007	UK	Retrospective review of referrals and first clinic appointment	2003–2005 (3 years)	All glaucoma referrals	1106	Compared to the diagnosis given to each patient on their first assessment at the HES	No glaucoma or OHT and discharged at first visit 531 (48%)
Bowling et al.^41^	2005	UK	Retrospective review of referrals and first clinic appointment	July 1994–June 2004 (10 years)	All glaucoma referrals	2506	Compared to the diagnosis given to each patient on their first assessment at the HES	Confirmed glaucoma 511 (20%) Glaucoma suspect 125 (5%) OHT 747 (30%) No glaucoma or OHT 1123 (45%) Discharged at first visit 1148 (45.3%)
Theodossiades et al.^19^	2004	UK	Prospective review of referrals (control arm)	June 2000–January 2001 (7 months)	All glaucoma referrals	119	Positive predictive value defined as a confirmed or suspected diagnosis of glaucoma, where ‘glaucoma’ encompasses open angle, closed angle and secondary glaucoma	Positive predictive value 55/119 (46.2%)

Abbreviations: CI, confidence interval; GOS, general ophthalmic services; HES, hospital eye service; OHT, ocular hypertension; PAC, primary angle closure; PACG, primary angle closure glaucoma; PACS, primary angle closure suspect; PI, peripheral iridectomy; SIGN, Scottish Intercollegiate Guidelines Network.

**TABLE 7:** Studies assessing referrals for cataract.

Study	Year	Country	Study design	Study period	Number of referrals	Measure of accuracy	Results
Canning et al.^43^	2022	Ireland	Retrospective audit of referrals	February 2021–February 2022 (1 year)	167	Listed for surgery after assessment by consultant ophthalmologist	114 (68.5%)
Do et al.^45^	2018	Australia	Retrospective audit of referral letters	August–September 2014 (2 months)	76	Listed for surgery/ surgery performed 12–15 months post‐referral	38 (50%)
Fung et al.^27^	2016	UK	Retrospective review of referrals	Backdated from 2014 (first quarter) until 1000 was reached (1991–2014)	26	Listed for surgery	21 (81%)
Davey et al.^4^	2011	UK	Retrospective audit of referral letters (Random sample)	2007–2008 (1 year)	Overall 61 Direct referral 8 Old GOS‐18: 32 New GOS‐18: 16 Letter 5	Listed for surgery	Overall, 45 (73.8%) Direct referral 8 (100%) Old GOS‐18: 23 (72%) New GOS‐18: 10 (63%) Letter 4 (80%)
Tattersall and Sullivan^46^	2008	UK	Retrospective audit of referral letters	August 2005 (2 weeks)	30	Clinical outcome after assessment by consultant ophthalmologist	23 (76.7%)
Lash et al.^13^	2006	UK	Prospective audit of referral letters	4 October–6 December 2004 (2 months)	351 Overall 162 GOS 18 143 Direct 46 Letters	Listed for surgery after assessment by consultant ophthalmologist	Overall, 272 (78%) Direct referral (83%) Referral letter (78%) GOS 18 (73%)
Lash^42^	2003	UK	Retrospective review of referrals	12 February–23 April 2001	163	Listed for surgery	77 (47%)

*Note*: GOS‐18 refers to the UK General Ophthalmic Services referral form.

**TABLE 8:** Study assessing referrals for neovascular age‐related macular degeneration (AMD).

Study	Year	Country	Study design	Study period	Number of optometrist referrals	Definition of accuracy	Accuracy
Muen and Hewick^18^	2011	UK	Prospective study of all optometry referrals using a rapid access referral form	December 2006–August 2009 (21 months)	54	Diagnosed with neovascular AMD by an ophthalmologist	20 (37%)

**TABLE 9:** Study assessing referrals for paediatric BV.

Study	Year	Country	Study design	Study period	Number of optometrist referrals	Definition of accuracy	Accuracy
Waters et al.^22^	2021	UK	Retrospective review of all referrals	March 2013–November 2017 (4 years, 9 months)	45	Condition confirmed during hospital consultation (match or partial match)	40 (88.9%)

Abbreviation: BV, binocular vision.

**TABLE 10:** Studies comparing referral accuracy of optometrists and General Medical Practitioners (GPs).

Study	Year	Country	Study design	Study period	Condition(s)	Number of optometrist referrals	Number of GP referrals	Definition of accuracy	Accuracy optometrists	Accuracy GPs
Waters et al.^22^	2021	UK	Retrospective review of all referrals	March 2013–November 2017 (4 years, 9 months)	Paediatric BV	45	54	Condition confirmed during hospital consultation (match or partial match)	40 (88.9%)	35 (65%)
Founti et al.^12^	2018	UK	Multicentre, prospective, observational, study	May 2013–March 2014 (10 months)	All glaucoma referrals	28	2	An outcome was defined as positive when the management plan was an intervention or active monitoring and as negative when the management plan was same‐day discharge	Positive 16 (57.1%, CI 24.6%–63%) Negative with same‐day discharge 12 (42.9%, CI 38.8%–75.4%)	Positive 1 (50%, CI 0%–100%) Negative with same‐day discharge 1 (50%, CI 0–100%)
Nari et al.^39^	2017	Canada	Retrospective review of referrals	17 January 2011 to 17 July 2011 (6 months)	Acute eye disease	309	102	Agreement with the final diagnosis	Correct 166 (54%) Incorrect 111 (36%) Non‐specific 18 (6%) Not yet diagnosed 12 (4%) Baseline 1 (<1%)	Correct 34 (33%) Incorrect 33 (32%) Non‐specific 27 (26%) Not yet diagnosed 4 (4%) Baseline 45 (4%)
Davey et al.^26^	2016	UK	Retrospective review of referrals	December 2007–December 2008 (1 year)	All ocular conditions	392 366 qualified 26 pre‐registration	131	True positives: Ophthalmologist confirmed condition/pathology. Discharge was not solely influenced by clinical techniques that were not currently commonly available to the referring practitioner Diagnostic agreement: Referring Diagnosis agrees with hospital	True positive: 361 (71%) Diagnostic agreement: 244 (67%)	True positive: 127 (97%) Diagnostic agreement: 73 (56%)
Fung et al.^27^	2016	UK	Retrospective review of referrals	Backdated from 2014 (first quarter) until 1000 were reached (1991–2014)	All ocular conditions	569	143	True positive: patients not being discharged from HES with a ‘normal vision’ diagnosis Diagnostic agreement: Concordance in referred condition and diagnosed condition at HES between optometrists and ophthalmologists	True positive: 93.8% Diagnostic agreement: 76.1%	True positive: 92.3% Diagnostic agreement: 67.2%
Jackson^34^	2009	Australia	Retrospective review of referrals from two hospitals	Alexandra Hospital 18 April–25 October 2006 (6 months 7 days) Royal Brisbane and Women's Hospital 1 July–30 September 2006 (3 months)	Acute eye disease	114	535	Agreement with the diagnosis made in the ophthalmology department	55/114 (48.2%)	192/535 (35.9%)
Pierscionek et al.^28^	2009	UK	Retrospective review of referrals	January–March 2007 (3 months)	All ocular conditions	323	243	True positive: Patient diagnosed as anything other than ‘no abnormality detected by ophthalmologist’ Diagnostic agreement: Referral diagnosis compared to the final diagnosis made by ophthalmologist	True positive: 302 (93.5%) Diagnostic agreement: 225 (69.7%)	True positive: 225 (92.6%) Diagnostic agreement: 160 (65.8%)

Abbreviations: BV, binocular vision abnormality; CI, confidence intervals; HES, hospital eye service.

**TABLE 11:** Comparison of diagnostic agreement accuracy for optometrists versus general medical practitioners (GPs).

Study	Agreement optometrists (%)	Agreement GPs (%)	Difference (%)	
Waters et al.^22^	88.9	65	23.9	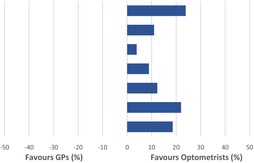
Davey et al.^26^	67	56	11	
Pierscionek et al.^28^	69.7	65.8	3.9	
Fung et al.^27^	76.1	67.2	8.9	
Jackson^34^	48.2	35.9	12.3	
Nari et al.^39^	54	33	22	
Weighted average	67.5	48.9	18.6	

**TABLE 12:** Studies assessing the factors affecting false‐positive (FP) referral rates.

Study	Year	Country	Study design	Study period	Number of optometrists/referrals	Factors assessed	Definition of accuracy	Accuracy	Factors
Parkins et al.^20^	2018	UK	Online vignettes	6 months	60 Optometrists 31 Qualified 18 Newly qualified 11 Pre‐registration	Years of Experience CET over 6 months	For each clinical vignette, Optometrists indicated what tests they would perform, their management decision, reason for decision and additional questions. Scoring was determined by an expert panel and participants' performance was compared to an expert	—	No correlation between change in score and CET points over the 6 months (*r* = 0.17, *p* = 0.37) No correlation between the change in score and the number of peer discussion sessions undertaken (*r* = 0.24, *p* = 0.90) Significant negative correlation between the number of referrals made by each practitioner and their time since qualification (*r*_s_ = 0.39, *p* = 0.005)
Davey et al.^26^	2016	UK	Retrospective review of referrals	December 2007–December 2008 (12 months)	366 referrals made by qualified optometrists	Gender Type of practice (multiple vs. independent) Years since professional registration Condition	False‐positive referral: Ophthalmologist discharged the patient due to the absence of significant ocular pathology OR Ophthalmologist diagnosed the patient with, or was suspicious of, pathology that was unrelated to the diagnosis given or implied by the optometrist. Decisions were not influenced solely by clinical techniques that were not currently commonly available to the optometrist	Optometrist (*n* = 366) 105 (29%) Female optometrists (*n* = 122) 47 (39%) Male optometrists (*n* = 159) 36 (23%) Multiple optical practice (*n* = 206) 74 (36%) Independent optical practice (*n* = 169) 38 (22%) Females in multiple practice (*n* = 82) 36 (44%) Females in independent practice (*n* = 40) 11 (28%) Males in multiple practice (*n* = 68) 21 (31%) Males in independent practice (*n* = 91) 15 (16%)	Females vs. Males (*p* = 0.008) Controlled for years since registration (*p* = 0.03) Controlled for years since registration and practice type (*p* = 0.07) Independent versus multiple (*p* = 0.005) Controlled for years since registration (*p* = 0.20) Controlled for gender and years since registration (*p* = 0.38) Condition (*p* = 0.046) (least to most FPs) 1. lens, 2. lids, lashes, 3. glaucoma, 4. everything else, 5. visual disturbance/other Proportion of FPs decreases by 6.2% per year since qualification (*p* < 0.001)

Abbreviation: CET, continuing education and training.

When reviewing the optometrists' referral accuracy literature, it was clear that there were several different focuses, mainly on a specific ocular condition or group of conditions. We recognise that different ocular conditions vary in prevalence (meaning optometrists' familiarity with the condition varies), referral urgency and available treatment options, so we grouped studies based on conditions to allow clear comparison. Other studies looked at referrals in general and/or factors that may contribute to a higher rate of inaccuracy such as referral source; these studies were also grouped based on their focus. The following sections discuss each of these groups, with some studies being allocated to more than one group. We begin by discussing studies that assessed referrals for multiple eye conditions before addressing specific eye conditions covered in the literature. We then compared the referral accuracy of optometrists with general medical practitioners (GPs) before lastly discussing optometrist factors that may affect their referrals.

### General optometric referrals

Seven studies assessed the accuracy of referrals for all ocular conditions by optometrists and are summarised in Table 4. One study, where the referrals assessed were used as a control group for a piloted new referral pathway, reported that of the referrals reaching the HES, 90% were deemed to require HES assessment by six ophthalmology consultants retrospectively reviewing the referrals and the outcomes of the initial HES appointment.^7^ Four studies assessed agreement between referral diagnosis and the diagnosis given at the first HES visit^15^, ^26^, ^27^, ^28^ and reported an agreement of between 67%^26^ and 76%.^28^ Of these four studies, three also reported the true positive rate. Two of the studies defined this as the patient having an abnormality and, thus, not being discharged on the first visit and reported true positive rates of 93.5%^28^ and 93.8%.^27^ The third study^26^ used a different definition for a true positive whereby the ophthalmologist's decision to discharge must not have been solely influenced by clinical techniques that were not commonly available to the referring practitioner and unexpectedly reported a lower true positive rate of 71%. Two studies from the same research group^29^, ^30^ measured referral accuracy through researchers assessing different aspects of the referrals. They reported that referrals were to an appropriate professional standard for 90%–100% of referrals across 6 dyads of optometry practices paired with a hospital eye department. The referral was necessary in 90.8%–97.5% of instances and was accurate in 81.1%–97.5%.^29^, ^30^ It can, therefore, be argued from that study that optometrists in the UK perform well in the identification of cases requiring referral overall. However, that study examined dyads with good levels of communication between the optometric practice and the hospital eye department and noted that poorly performing optometry practices would be less likely to participate in a study that scrutinised their performance.

### Referrals for emergency eye conditions

Another important aspect of the accuracy of referrals is not just assessing whether a referral was necessary but also whether the suggested urgency of referral was appropriate. Many patients who visit emergency eye departments have been referred by their optometrist, with this proportion having previously been reported as up to 12% of eye casualty attendances.^31^, ^32^ These referrals are important to assess as emergency departments are well known for having long waiting times, and patients must attend an appointment either physically, or more recently remotely, to be triaged.^33^

Four studies assessed the accuracy of referrals of emergency eye conditions from optometrists, which are summarised in Table 5. For the studies that reported the percentage of ‘correct’ diagnoses in referrals, the optometrists' accuracy ranged from 48.2%^34^ to 60%.^16^ The study measuring accuracy using kappa statistics^25^ reported a kappa agreement across a range of different eye conditions of good (0.59) for neuro‐ophthalmology to excellent (0.87) for anterior segment conditions. In one study, carried out in Canada, 21.1% of emergency referrals from optometrists were determined to require ‘urgent’ HES attention,^16^ defined as ‘should be seen that day’. In that study, semi‐urgent was defined as ‘should be seen within 1 day of referral’ (47.4%), with the remaining 31.6% of patients deemed non‐urgent (could be seen more than 1 day after referral).

### Referrals for glaucoma

Glaucoma subspecialty appointments are responsible for approximately a fifth of all HES workload, with an expected increase in incidence of the disease in the coming years.^35^ Glaucoma suspects are typically monitored over a period of time for progression at regular appointments before discharge or decision to treat, and those patients diagnosed with glaucoma require lifelong clinical follow up.^36^ This creates an accumulative workload for glaucoma clinics to manage, to which unnecessary referrals into the service further contribute. It is, therefore, important that referrals for suspected glaucoma are accurate and appropriate.

Overall, 11 studies assessed the accuracy of glaucoma referrals into the HES from optometric practice and are summarised in Table 6. Ten of the studies compared optometrist referrals to the diagnosis determined by an ophthalmologist at the patient's first visit to the HES and one after at least two visits; however, the studies used different definitions for measuring the accuracy of referrals. One study determined an outcome as positive based on a clinical diagnosis of primary angle closure suspect (PACS), primary angle closure (PAC) or primary angle closure glaucoma (PACG) according to the International Society of Geographical and Epidemiological Ophthalmology classification.^21^ This was the only study of the 11 to focus specifically on closed‐angle glaucoma. When considering the percentage of patients discharged at the first visit, studies reported a range from 16.7%^23^ to 48%.^37^ One study reported a higher discharge value of 62.6%^14^ but this was after at least two visits to the HES. Two studies assessed the accuracy of optometrist referrals into the HES pre and post new community optometry referral guidelines.^23^, ^24^ Both of these studies took place in Scotland and reported a decrease in the first visit discharge rate (FVDR) after new General Ophthalmic Services (GOS) contracts (43.2% old GOS to 16.7% new GOS, *p* = 0.004)^23^ and Scottish Intercollegiate Guidelines Network (SIGN) guidelines (29.2% pre‐SIGN to 19.4% post‐SIGN).^24^

One of the reviewed studies reported an unusual finding.^38^ The study carried out in the Republic of Ireland reported that on first assessment in the HES, 67% of patients were classified as normal; however, only 35% were discharged. This finding may have been due to patients being seen within a private hospital, meaning the consultant would have more flexibility to bring patients back for another review even if considered ‘normal’ at their first visit. Due to its progressive nature, glaucoma can be difficult to diagnose based on one examination and the consultant may have wanted to review some patients again, especially if possessing disease risk factors. The paper focussed on the comparison of non‐contact tonometry measures of intraocular pressure (IOP) on referral with Goldmann applanation tonometry at the first HES visit. Large differences between the two IOP measures may have been another prompt to review patients again and test for fluctuations in IOP such as diurnal variations.

### Referrals for cataract

Cataract referrals make up the largest proportion of referrals from primary care to secondary care in the UK.^26^, ^27^, ^28^ Investigating the accuracy of these referrals is essential to explore the potential strain that these initial numbers put on the HES. However, the method of assessing the accuracy of cataract referrals is different from other common ocular conditions as referrals should only be made to the HES to initiate listing for surgery. Thus, the seven studies evaluated in this review assessed accuracy of referrals from optometrists based on whether patients had been listed after being seen within the HES and are summarised in Table 7. The listing rate ranged from 47%^42^ to 81%^27^ for referrals overall, with a very recent study from the west of Ireland reporting an intermediate value (68.5%).^43^ Two studies separated cataract referrals into the method of referral.^13^, ^26^ In both studies, the listing rate increased when a direct referral was made from the optometrist to the HES by either 83%^13^ or 100%.^26^ In both studies, the lowest rates came from referrals that used the GOS18 forms. For Lash et al.'s study, this rate was 73%. For Davey et al.'s study this listing rate was 63% for the ‘new’ GOS‐18 forms and 72% for ‘old’ GOS‐18 forms.

### Referrals for neovascular AMD

Only one paper focussed on optometric referrals for neovascular AMD (Table 8). This study, carried out in the UK, used a prospective study design over a 21‐month period to evaluate optometric referrals to the HES, specifically for neovascular AMD using a rapid access referral form.^18^ This study assessed 54 referrals and found that only 20 (37%) were confirmed as having neovascular AMD. Additionally, this study assessed agreement of optometrist referrals with an ophthalmologist with respect to the specific clinical signs reported on referral. The agreement for retinal haemorrhage was 83.3%, for exudates 66.7%, for drusen 51.9% and for subretinal fluid 44.4%. The most common conditions that the optometrists had misdiagnosed as neovascular AMD were dry AMD (18.5%), epi‐retinal membrane (9.3%), branch retinal vein occlusion (7.4%) and central serous chorioretinopathy (7.4%).

### Paediatric referrals

Optometrists play an important role in the screening of children for reduced vision and possible binocular vision (BV) abnormalities, and optometry paediatric screening in the UK may be preferred over visiting a GP practice, due to the limited speciality knowledge of GPs.^44^ Only one study assessed the accuracy of optometrists' referrals of paediatric patients (Table 9).^22^ This retrospective analysis was mainly focussed on the accuracy of GP referrals but also reported separately the accuracy of referrals initiated by optometrists. This study of 45 optometrist referrals for children with suspect BV abnormalities found that 88.9% of referrals either fully or partially matched the diagnosis made by an ophthalmologist in the HES. The accuracy of diagnosis also increased with patient age, with 0% (*n* = 1) accuracy for patients 0–2 years old, 87% (*n* = 23) accuracy for patients 3–6 years old and 90% (*n* = 21) accuracy for patients 7–13 years old. However, the link between age and referral accuracy was not statistically significant (*p* = 0.06).

### Comparison of optometrists with GPs

Assessing the accuracy of referrals between optometrists and GPs is important to determine which of these practitioners manage specific eye conditions more appropriately. Seven of the studies assessed the accuracy of optometrist referrals in comparison with GP referrals (Table 10). Of these, three assessed the accuracy of referrals for all eye conditions.^26^, ^27^, ^28^ All three studies reported higher diagnostic accuracy for optometrists (67% vs. 56%, 69.7% vs. 65.8% and 76.1% vs. 67.2%). When assessing the true positive rate, two studies^27^, ^28^ reported a higher rate for optometrists when defining a true positive as a referral whereby an abnormality was present, even if the referral findings/diagnosis did not match the HES report (93.5% vs. 92.6% and 93.8% vs. 92.3%). The third study^26^ reported a higher true positive rate for GPs (96% vs. 71%) but used a different definition for a true positive whereby the ophthalmologist's decision to discharge must not have been solely influenced by clinical techniques that were not commonly available to the referring practitioner. These commonly available techniques were not defined so it was unclear how much they differed between practitioners. Two studies assessed the accuracy of referrals for acute eye conditions.^34^, ^39^ Both reported a higher accuracy of optometrist referrals (48.2% and 54%) compared with GP referrals (35.9% and 33%). One study assessed the accuracy of referrals for paediatric BV conditions.^22^ This study defined an accurate referral as a full or partial match to the diagnosis made at the first visit to the HES, where a partial match was not clearly defined, and reported significantly higher accuracy of optometrist referrals (88.9%) compared with GP referrals (65%; *p* = 0.01). One study assessed the accuracy of referrals for suspected glaucoma^12^ and reported a higher accuracy of referral for optometrists, defined as a positive outcome when the management plan was an intervention or active monitoring. Optometrist referrals were positive for 57.1% compared with 50% of GP referrals. However, this study assessed a very small number of referrals, with only two referrals coming from GPs.

Table 11 represents a summary of the accuracy of referrals from optometrists and GPs reported when using agreement with an ophthalmologist at the HES appointment as the measure of accuracy. A weighted average accuracy was calculated for both optometrists and GPs by accounting for the sample size used in each study, that is, the reported percentage accuracy was multiplied by the sample size for each study before adding these results together. The total was then divided by the total sample size of all of the six studies. Overall, optometrists had an accuracy rate that was 18.6% higher than GPs for diagnostic agreement.

### Optometrist factors affecting the accuracy of referrals

To work towards improving the accuracy of optometrist referrals, it is important to assess the possible factors which may be influencing referral decisions. Two studies, both carried out in the UK, assessed the optometrist factors that may influence the accuracy of referrals (Table 12). One of the investigations was an online vignette study, whereby optometrists indicated their management decision and reason for the decision.^20^ This study assessed years of clinical experience and continuing education and training (CET) points completed over 6 months as factors and reported no correlation between the change in score and CET points over the 6 months (*r* = 0.17, *p* = 0.37); there was no correlation between the change in score and the number of peer discussion sessions undertaken (*r* = 0.24, *p* = 0.90). However, the type of CET training undertaken was not standardised. There was a significant negative correlation between the number of referrals made by practitioners and their time since qualification (*r*_s_ = 0.39, *p* = 0.005). However, although initiating more false‐positive referrals, it is unclear how the level of experience may affect false‐negative referrals. The clinical vignette study^20^ reported that three participants with over 20 years of experience only referred five cases despite six being chosen as certain referrals in the study design. In comparison, the seven participants that referred ≥10 cases all had at most 4 years of experience. Eight cases were also chosen as ‘grey area’ cases where there was no definite correct answer, so although less experienced practitioners referred more cases, it was not clear whether that meant they were incorrect. The second study was a retrospective review of referrals into the HES.^26^ They reported that female optometrists made significantly more false‐positive referrals than males (39% vs. 23%, *p* = 0.008), and this significant difference was still present when years since registration was controlled for (*p* = 0.03). The proportion of false‐positives decreased by 6.2% per year since registration (*p* < 0.001). There was a significantly higher proportion of false‐positive referrals from multiple practices compared with independent practices (*p* = 0.005), but this value became insignificant when controlling for years since registration (*p* = 0.20). The proportion of false‐positive referrals also had a significant link to the type of condition referred (*p* = 0.046), with referrals for lids/lashes being the most accurate and referrals for visual disturbance/other being the least accurate.

## DISCUSSION

In this section, we discuss the main findings from the reviewed studies and their possible implications based on four core themes:

1. Condition‐based referral accuracy.
2. Optometrist factors affecting referral accuracy.
3. Missing information in the literature.
4. Enhanced referral schemes.

The first two themes were identified by comparing the methodology and outcomes across all the reviewed studies and linked directly to the objectives of the review. The third and fourth themes were identified based on knowledge of current practice independent of the studies meeting the inclusion criteria for this review. The third theme was specifically shaped by information that we expected to have been included in the literature. We discuss these four themes separately, with some also containing subthemes.

### Condition‐based referral accuracy

It was evident from our review that there is variability in the accuracy of referrals depending on the type of eye condition(s) being referred, with one study that compared the accuracy of all referrals based on condition reporting a just significant effect of condition group on the level of false‐positives^26^ (*p* = 0.046). Overall, from our review, optometrists' referral accuracy based on the diagnostic agreement with specialists in the HES varied across eye conditions. This variation is not surprising, as the frequency with which different conditions are encountered in primary care varies, meaning optometrists may feel more confident in their examination of commonly encountered conditions such as cataract compared with, for example, suspected neuro‐ophthalmological disease. Additionally, the risk to the patient of delaying intervention for different conditions varies. Using the same examples, delaying the identification and treatment of a neuro‐ophthalmological condition would typically pose a much higher risk to the patient's sight/life than a cataract. Of note, the range for the accuracy of referrals for suspected emergency ocular conditions as a whole was lower than for other conditions that were covered in detail, with only 21.1% of emergency referrals considered to require urgent attention in one study.^16^ This may indicate that optometrists are erring on the side of caution for conditions they consider potentially urgent. However, it also highlights ambiguity in the terms used to describe different referral urgencies. In that study, ‘semi‐urgent’ was defined as still needing to be seen within 1 day of referral. In comparison, the College of Optometrists ‘Urgency of Referrals’ guidelines define this same timeframe as an ‘emergency’.^47^ Thus, the proportion of referrals appropriately directed to an emergency department rather than via a routine pathway appears higher than the 21.1% which were determined to be ‘urgent’. In that same study, the vague definition for ‘nonurgent’ (could be seen greater than 1 day after referral) also meant that referrals requiring review from a range of 2 days post‐referral up to a routine referral timeline such as 3 months or longer could be classed as ‘nonurgent’.

As the accuracy for conditions such as neovascular AMD and paediatric BV were only addressed by one study in our review, it was difficult to draw conclusions for these conditions. It is somewhat surprising that our literature search found only one study focusing on the accuracy of referrals for AMD, considering that this is the most frequent cause of visual impairment in developed countries and that distinguishing the ‘wet’ from the ‘dry’ form is essential for determining which patients require treatment.

The difference in referral accuracy across ocular conditions also makes it difficult to draw conclusions from the studies comparing the accuracy of referrals from GPs and optometrists, as the practitioners largely refer to different eye conditions. One of the reviewed studies reported that 40% of GP referrals were for disorders of the lacrimal system, eyelids and orbit, whereas referrals for the same group of conditions made up less than 5% of optometrist referrals.^27^ In comparison, the most referred condition from optometrists was disorders of the lens, which made up 20% of optometrist referrals but only around 7% of GP referrals. This suggests that patients report more commonly to GPs for conditions of the lids/lashes and lacrimal system. However, it may also suggest that GPs are more comfortable referring these conditions themselves but may send patients to optometrists for assessment of other suspected ocular abnormalities, perhaps due to the lack of available ophthalmic techniques and specialised training in general practice.

### Referrals for cataracts

One condition encountered frequently in primary care practice is cataracts, which are typically easily identified during an ocular health check. The referral accuracy for cataracts was covered in detail by the studies reviewed. As cataracts are most commonly age‐related and slowly progressing, they should be monitored in primary care until a referral to the HES is necessary to initiate listing for surgery. Thus, the studies evaluated in this review assessed the accuracy of referrals from optometrists based on whether the patients had been listed for surgery, as a surrogate measure for whether a referral to the HES was appropriate. Although optometrists are competent in identifying cataracts on examination and reported referral accuracy was reasonable, the fact that listing rates were not nearing 100% for typical referral routes means many patients are being referred before surgery is indicated. The ‘Action on Cataracts’ government guidance in the UK^48^ stated that cataract referrals should be based on reduced visual acuity, impaired lifestyle and the willingness of the patient to have surgery, to avoid unnecessary referrals. In the studies carried out in the UK, it was reported that the main reason for patients not being listed for surgery was due to them not being symptomatic.^13^, ^46^ This suggests that a number of patients who are not yet symptomatic are being referred unnecessarily, perhaps due to optometrists either not asking the correct symptoms and lifestyle questions prior to referral or that optometrists' thresholds with respect to symptoms requiring surgery are lower than those of the ophthalmologists. This of course would require further assessment.

### Referrals for glaucoma

Another condition covered in detail by the reviewed studies was suspected glaucoma. Although encountered in primary care more often than rarer optic neuropathies such as optic neuritis, it is still seen infrequently in primary care practice. The suboptimal referral accuracy reported is not surprising, as glaucoma diagnosis and detection can be very tricky, particularly in the early stages of disease and partly due to its characteristically progressive nature. As previously mentioned, is is also rare for optometrists to receive feedback about the outcomes from their referrals, making it difficult to learn from previous patient encounters.

Normal physiological variations in optic nerve morphology can make the identification of a glaucomatous optic nerve difficult and visual field testing and IOP measurements can be variable, with repeated testing advised for many cases where abnormal results are found. The best practice for optic disc evaluation would be a stereoscopic view through a dilated pupil, but it may be impractical for optometrists working in busy practice to perform dilation on all glaucoma suspects. Optometrists practising in the UK have previously reported that they were constrained by time and are required to see a patient every 20–30 min.^49^ This means that additional tests such as repeated visual fields, Goldmann tonometry and/or a dilated fundus examination would be virtually impossible in the time available.

Although the College of Optometrists clinical management guidelines provides clear advice for the referral of a range of suspect ocular conditions; for glaucoma, detailed guidelines in relation to a risk assessment based on specific clinical findings and patient history are lacking in England. The latest UK National Institute for Health and Care Excellence (NICE) referral guidelines for glaucoma case‐finding in primary care^50^ give recommendations for tests that should be completed and when tests should be repeated. However, these guidelines are non‐specific and recommend that those planning eye care services should consider commissioning referral filtering services such as enhanced refinement schemes, where more detailed assessments are carried out. The results from a reviewed study carried out in Scotland suggest that a change in primary care guidelines, specific to Scotland, has improved the accuracy of glaucoma referrals. Since the introduction of a new GOS contract in 2006, which introduced supplementary examinations such as central corneal thickness measurement, there was a consensus that specific referral guidelines should be set out.^51^ This led to the introduction of the Scottish Intercollegiate Guidelines Network (SIGN) guideline 144 in March 2015,^52^ which included more detailed advice in relation to interpreting results of numerous glaucoma screening tests in combination. Results from reviewed studies have suggested a positive impact of both the GOS contract^23^ and the new SIGN guidelines,^24^ suggesting that similar guidelines, if implemented in other countries/regions may aid optometrists in making better referral decisions.

Particularly for the reviewed studies assessing glaucoma referrals, we must also consider the time periods from which the referral samples were assessed. This is due to the publication of changing referral guidelines in the UK during the past 20 years. In December 2009, the College of Optometrists released guidelines, which advised optometrists to refer patients with an IOP > 21 mmHg, even in the presence of normal optic discs and visual fields, stating that practitioners could leave themselves ‘legally exposed’ if they failed to do so. This guidance may explain why two studies carried out in 2010 and 2011^14^, ^40^ both found that when referral was based on one measure alone, IOP was the most common, with this being the case in 44%^14^ and 43% of patients.^40^ This contradicted an earlier study prior to the 2009 guidelines^37^ which reported that 65.5%–74.3% of referrals were made based on optic disc appearance alone. It must also be noted that the NICE and College of Optometrists guidelines again changed in 2017 and recommended that referral based only on IOP should be when IOP is ≥24 mmHg using Goldmann‐type applanation tonometry; none of the studies identified in our review used samples taken after this new guidance was published. Since its introduction, the number of referrals based on IOP findings alone, as well as the proportion of false‐positive glaucoma referrals may have reduced, due to an increase in the IOP threshold guidance for referral.

### Definitions for referral accuracy

As well as there being a range in referral accuracy between conditions, there was also variability between studies reporting the referral accuracy for the same condition. When reviewing the studies, it was evident that there was significant variation in the classifications used when determining whether a referral from primary care optometrists was accurate. This created some difficulty when interpreting and comparing the results reported and appeared to be a contributing factor as to why differences in referral accuracy within the same eye condition were reported. One approach used by many of the studies was to assess whether optometrists' referral diagnosis agreed with the ophthalmological diagnosis made on assessment at the HES. Comparing the diagnosis made by an optometrist in primary care with that of an ophthalmologist in the HES can be problematic as optometrists are generally more limited with respect to the equipment and diagnostic aids available to them. Additionally, many optometrists carry out sight tests alone, in busy clinics, without access to specialist opinion, and often rely on their individual clinical judgement to decide on a most likely diagnosis and management decision. Primary care optometrists can therefore be considered overall as more ‘generalist’ in their knowledge and experience. In comparison, clinicians in the HES tend to be more specialised, often receiving additional training and having significantly more experience with specific eye conditions. They often have advanced diagnostic techniques available to them and other specialists to ask for advice or opinions on complex clinical cases.

It can therefore be argued that a more appropriate assessment of the accuracy of referrals is to determine whether a referred patient required assessment in the HES or not, regardless of whether the referral diagnosis matched the diagnosis made during the HES appointment. This method of assessing referral accuracy specifically focusses on the rate of ‘false‐positive’ referrals made and was used by a number of reviewed studies by identifying which patients required onward referral and could not be safely managed in primary care. The General Optical Council (GOC) standards of practice guidelines state that optometrists should ‘recognise and work within the limits of your scope of practice’ and ‘be able to identify when you need to refer a patient in the interests of the patient's health and safety, and make appropriate referrals’;^5^ thus, optometrists should refer any condition that they feel unable to manage in practice. One may argue that tentative diagnoses do not need to be completely accurate but that the referral needs to be appropriate.

We can also argue that to evaluate the accuracy of referrals fully, the false‐negative rate should also be assessed. This measure would identify the number of referrals that require review in the HES but were not referred by optometrists. Only one study reported a false‐negative referral rate and focussed specifically on narrow anterior chamber angle identification,^21^ with their population consisting only of patients referred for suspected glaucoma, which is not representative of all patients tested in primary care. Other studies outside this review have also successfully assessed the false‐negative referrals generated within referral triage pathways such as glaucoma referral refinement,^53^, ^54^, ^55^ and assessed false‐negatives within management decisions made as part of the COVID urgent eye care scheme.^56^ We recognise that false‐negative referral rate from eye examinations performed in routine primary eye care practice would be difficult to measure, as it would require a secondary assessment of unreferred patients and is unlikely to be feasible; however, it is important to consider as a shortcoming of the reviewed studies.

### Optometrist factors

The reviewed studies identified several factors, which may contribute to the accuracy of referrals made by optometrists. Firstly, it is not surprising that for both studies assessing optometrist factors, a shorter time since qualification was associated with a significantly higher number of referrals made and lower referral accuracy.^20^, ^26^ Although significantly more false‐positive referrals were made from multiple practices compared with independent practices,^26^ this appeared to be explained by multiple practices employing optometrists with fewer years of experience. In the early stages of qualification, optometrists are likely to be more cautious with their clinical decision‐making, especially when assessing eye conditions that they are not familiar with. Through gaining experience and learning from previous patient encounters, optometrists are likely to become more confident with their clinical assessment and ability to manage patients in primary care.

In a retrospective study,^26^ the results also suggest that female optometrists were significantly more likely to make false‐positive referrals compared with male optometrists, which remained the case when years since registration was controlled for. The authors suggest that this finding may be explained by ‘years since registration’ not being an accurate representation of clinical experience, particularly for females. Females are more likely to take career breaks for maternity leave or to work part‐time due to care commitments,^57^ and these interruptions can affect the continuity of practice and training. However, previous studies of other clinicians, such as GPs, have also found evidence of differences in clinical decision‐making between males and females. One study found that female primary care physicians were more likely to refer patients^58^ and other studies have reported more aggressive disease screening in patients of female physicians, irrespective of the patient's gender.^59^, ^60^ Although recent studies are lacking, these may indicate a more cautious management approach by females, which could lead to a higher number of false‐positive referrals. Again, however, there was no available measure of false‐negative cases and gender as a factor was reported by one study only.

### Missing information in the literature

Another theme formed from our analysis was that the literature was lacking in certain topics and/or backgrounds. Of note, 23 of the 31 studies reviewed were carried out in the UK. This means that our findings apply primarily to UK optometry practice. A smaller number of studies were carried out in Canada (*n* = 2), Australia (*n* = 3), Norway (*n* = 1) and the Republic of Ireland (*n* = 2), but there was overall little diversity. This is likely to be due partly to our inclusion criteria excluding studies that were not published in English; however, it may also be due to large differences in eyecare systems across the world, with optometrists playing varied roles in countries with different scopes of practice. Even within the UK, eyecare pathways and local guidelines can differ considerably between regions. Thus, we recognise that results from the reviewed studies may not accurately represent the accuracy of referrals internationally or in the UK overall and may be specific to the regions in which they were carried out.

### No focus on ocular imaging

Another topic that was lacking in the reviewed literature was an examination of advanced ocular imaging, such as Ocular Coherence Tomography (OCT), and how its use may have affected the referrals being made from primary to secondary care. In recent years, there has been a dramatic increase in the use of advanced ocular imaging in UK primary care, especially since 2017, when Specsavers Opticians, the largest multiple practices in the UK, announced their investment in a multi‐million‐pound programme to introduce OCT imaging into each of its 900 practices.^61^ One might expect that the introduction of OCT scanning has increased the rate of false‐positive referrals for suspected retinal disease. This is because the detailed visualisation of retinal layers provided by OCT devices may identify benign changes in asymptomatic patients that appear as abnormalities and would otherwise be undetected. Conversely, the increased clinical information presented by OCT imaging is likely to have improved optometrists’ ability to detect subtle pathological features such as retinal fluid, and thus, detect more conditions requiring urgent referral such as choroidal neovascularistion.

A pilot study by Kern et al.,^62^ where primary care optometrists referred patients via a web‐based interface with retinal and OCT imaging included, found that after patients' data were reviewed virtually by a retinal specialist, 54 (52%) patients initially referred did not require specialist review. However, as this was a piloted system it does not represent the accuracy of referrals being made based on OCT imaging within the currently used referral pathways and did not meet our inclusion criteria for this review. Jindal et al.^6^ found that the use of OCT scans along with fundus imaging improved community optometrists' diagnostic sensitivity for both optic nerve and retinal abnormalities for clinical vignettes; however, this study only assessed diagnoses and not optometrist referral suggestions. Thus, during our literature review, we did not identify any studies assessing the effect of advanced ocular imaging on the accuracy of referrals in current practice and were unable to assess the effect this may have had in recent years.

### Enhanced referral schemes

Within the UK, an oversubscription to ophthalmic hospital services has led to interventions that attempt to improve referral accuracy and ultimately reduce the number of false‐positive referrals being seen in secondary care face‐to‐face clinics. Two of the reviewed studies also assessed a scheme for cataract referrals through an established direct referral system where accredited optometrists performed a dilated fundus examination, discuss cataract surgery with the patient and use a cataract‐specific proforma to achieve a higher level of referral quality. These studies reported the highest listing rates when the enhanced route was used of 83%^13^ and 100%^4^ compared with referrals via the GP through the standard referral pathway.

In some areas, asynchronous virtual review of optometric referrals carried out by ophthalmologists is also being used or has been trialled. This method aims to triage referrals virtually, and was reported in a pilot study to reduce the number of patients (for suspected retinal pathology) being seen face‐to‐face within the HES by 52%.^62^ Such pathways can improve two‐way communication between primary and secondary care and allow feedback to optometrists, which is significantly lacking within standard referral pathways.^63^ This feedback could help optometrists keep up to date with outcomes of patients they have previously referred and avoid unnecessary re‐referrals. It could also act as a learning aid, enabling them to make better management decisions if/when encountering similar cases in the future.

Another enhanced service scheme in place across different areas of the UK and Australia is glaucoma referral refinement. Referral refinement schemes have been successfully implemented in some areas and have been reported to improve the accuracy of glaucoma referrals^17^, ^53^, ^64^ as well as being potentially cost‐saving for the NHS^65^ and accepted by patients.^66^ A detailed evaluation of the success of the schemes discussed is beyond the scope of this review, as we aimed to assess referrals from optometrists using standard pathways, but are important to consider as when established, they are likely to have affected referrals into the HES.

### Clinical implications and conclusions

Based on the reviewed studies, although overall reasonable levels of accuracy were reported for general referrals, there was a large variation in referral accuracy across different ocular conditions. Recent studies are lacking, which means the effect of increased advanced imaging on the number and accuracy of primary care referrals requires further evaluation.

For glaucoma referrals, which were covered in the most detail in the papers reviewed, the rates of false‐positive and first‐visit discharge were suboptimal. This is important as glaucoma appointments are responsible for approximately a fifth of all HES workload, and make up a high proportion of referrals from optometric practice. Further development and increasing the uptake of refinement schemes for glaucoma referrals throughout the UK may help to reduce the number of unnecessary appointments. Referrals for cataract surgery make up the highest number of referrals from primary care optometric practice. Improved communication between optometrists and patients regarding visual symptoms and willingness for cataract surgery could improve listing rates and reduce waiting times.

Approaches have already been made to reduce the number of false‐positive referrals entering the HES, but with eyecare systems varying greatly across regions, it is difficult to determine the most efficient way to address the problem. The College of Optometrists clinical management guidelines provide clear advice for the referral of suspected ocular conditions. However, for glaucoma, specific guidelines in relation to a risk assessment based on specific clinical findings and patient history are lacking in England and may be a useful resource to improve the accuracy of referrals made from primary care.

Another approach is to focus on the widespread development of virtual referral pathways to reduce unnecessary face‐to‐face clinic time, reduce patient waiting times and anxiety, improve care and increase cost‐effectiveness. Additionally, virtual pathways would hopefully promote two‐way communication between primary and secondary care to encourage feedback on referrals, which would particularly benefit those optometrists with less experience to learn and improve the accuracy of their referrals.

Overall, based on this review, optometrists' referral accuracy can be considered suboptimal. However, it may be unreasonable to expect an optometrist working in primary care, with limited time and varied resources, to achieve high diagnostic accuracy. One could argue that optometrists are working within their scope of practice and that choosing the cautious option of referral is in the patients' best interests, especially when they feel uncertain of a diagnosis. Hospital eye clinics are overrun, and approaches should be made to improve referral accuracy as far as possible to reduce unnecessary face‐to‐face appointments.

## AUTHOR CONTRIBUTIONS

**Josie Carmichael:** Conceptualization (lead); data curation (lead); formal analysis (lead); writing – original draft (lead). **Sarah Abdi:** Data curation (supporting); writing – review and editing (equal). **Konstantinos Balaskas:** Funding acquisition (equal); supervision (supporting); writing – review and editing (equal). **Enrico Costanza:** Supervision (supporting); writing – review and editing (equal). **Ann Blandford:** Conceptualization (supporting); funding acquisition (equal); supervision (lead); writing – review and editing (equal).

## CONFLICT OF INTEREST STATEMENT

The authors have no conflicts of interest to declare.
